# Research trends in olanzapine in treating schizophrenia from 2010 to 2026: A bibliometric analysis based on the Web of Science Core Collection

**DOI:** 10.1097/MD.0000000000050747

**Published:** 2026-09-25

**Authors:** Na Shu, Lin Wang, Ling Xin Wang, Yan Gou, Min Zhao, Hai Ming Tang

**Affiliations:** a Department of Pharmacy, Luzhou Mental Hospital, Luzhou, China; b Department of Geriatric Psychiatry, Luzhou Mental Hospital, Luzhou, China.

**Keywords:** bibliometrics, CiteSpace, knowledge mapping, olanzapine, schizophrenia

## Abstract

**Background:**

Schizophrenia is a severe chronic mental disorder, and olanzapine is widely used in its treatment. However, the expanding and heterogeneous literature on olanzapine in schizophrenia has not been comprehensively characterized from a bibliometric perspective. Therefore, this study aimed to systematically characterize the research landscape, hotspots, emerging frontiers, and knowledge evolution of olanzapine in the treatment of schizophrenia from 2010 to 2026.

**Methods:**

Literature on olanzapine for the treatment of schizophrenia published between January 1, 2010, and March 29, 2026, was retrieved from the Web of Science Core Collection. CiteSpace (version 6.4.R2) and the Bibliometrix R package were used for bibliometric and visual analyses. Multiple bibliometric characteristics were analyzed, including publication trends, national and institutional distributions, core authors, journal distributions, co-citation of references, co-occurrence, clustering, keyword burst detection, and disciplinary biclustering.

**Results:**

A total of 3943 valid publications were included. Annual publication volume generally increased over the study period, despite periodic minor fluctuations. The United States, China, and the United Kingdom accounted for the largest shares of publication output. From a bibliometric perspective, University College London stood out as a hub node within the collaboration network. Correll CU and Leucht S represented highly productive contributors. *Schizophrenia Research* was the most productive journal in terms of publication output. Keyword analysis indicated that the research focus gradually shifted from evaluation of drug efficacy and dosage-form development to metabolic risk management, personalized treatment, and real-world clinical research. Recent research primarily focused on population-based cohort studies, comparative evaluations of treatment efficacy, clinical practice guidelines, and treatment-related risk assessment.

**Conclusion:**

Research on olanzapine for the treatment of schizophrenia is ongoing. Current studies have shifted from efficacy verification to safety monitoring, with growing emphasis on population-based individualized dose adjustment, metabolic risk management, and large-sample comparative clinical studies for clinical guideline optimization.

## 1. Introduction

Schizophrenia is a severe chronic mental disorder primarily characterized by impairments in cognition, perception, emotion, and social functioning, which can result in a significant disease burden and long-term functional disability.^[1,2]^ Pharmacotherapy is the cornerstone of standardized management for schizophrenia. Second-generation antipsychotics (SGAs) offer greater safety than traditional antipsychotics and are recommended as first-line treatments.^[3]^ Olanzapine is one of the most widely used SGAs in clinical practice; it demonstrates clear efficacy against both positive and negative symptoms and is commonly used for acute intervention and long-term relapse prevention.^[4–6]^ However, dose-dependent metabolic adverse effects limit its clinical use, including weight gain, hyperglycemia, and dyslipidemia, which may reduce treatment adherence and increase the risk of cardiovascular disease.^[7–13]^ In recent years, several new strategies – such as a fixed-dose combination of olanzapine and salmeterol, as well as metabolic interventions – have emerged to optimize its metabolic safety.^[14–16]^

Over the past few decades, clinical research on olanzapine for the treatment of schizophrenia has grown rapidly, yielding a vast body of heterogeneous evidence spanning multiple areas, such as efficacy, safety, and pharmacological mechanisms.^[17,18]^ Traditional narrative reviews and systematic reviews have primarily focused on summarizing specific clinical outcomes or mechanisms, but they struggle to systematically describe the overall knowledge structure, research collaboration networks, and dynamic evolution of research in this field.

Some existing bibliometric studies have analyzed the overall research landscape of antipsychotic drugs.^[19–21]^ However, most of these studies either included all SGAs or analyzed research output from specific regions only, lacking a timely, in-depth analysis specifically targeting the olanzapine–schizophrenia domain. Furthermore, existing bibliometric evidence has failed to incorporate significant research advances from the past decade, such as long-acting injectables, novel combination formulations, and precision management of metabolic risks. Methodologically, previous studies have largely been limited to descriptive indicators, lacking comprehensive knowledge graph analyses of research hotspots, cutting-edge shifts, and disciplinary knowledge flows.

This study employed bibliometric analysis and knowledge-graph visualization methods to systematically review research literature on the use of olanzapine to treat schizophrenia published between January 1, 2010, and March 29, 2026, and indexed in the Web of Science Core Collection. This study aimed to clarify the distribution of key research contributors, the temporal evolution of research hotspots, and emerging trends, thereby providing a reference for future research planning and clinical investigations.

## 2. Methods

### 2.1. Data sources and search strategy

A literature search was conducted in the Web of Science Core Collection on March 29, 2026. Records published between January 1, 2010, and March 29, 2026, were considered eligible. The search strategy combined Topic (TS), Title (TI), Abstract (AB), and Author Keywords (AK) fields to maximize retrieval sensitivity and minimize the risk of missing relevant publications. All duplicate records were removed during screening. The full retrieval formula was as follows: (TS = olanzapine OR TI = olanzapine OR AB = olanzapine OR AK = olanzapine) AND (TS = schizophrenia OR TI = schizophrenia OR AB = schizophrenia OR AK = schizophrenia). Only original articles and reviews in English were included, while case reports, conference abstracts, letters, editorials, irrelevant records, and retracted papers were excluded. The full search details are presented in Table S1, Supplemental Digital Content 1.

### 2.2. Data preprocessing

Bibliographic records were downloaded from the Web of Science in plain-text format (full records and cited references) and CSV format, and saved as text files with filenames beginning with “download_*.” All records were imported into CiteSpace (version 6.4.R2; developed by Chaomei Chen at Drexel University) for format conversion, deduplication, and data cleaning, yielding a dataset suitable for subsequent analyses. Keywords were standardized by merging synonyms, removing meaningless or overly general terms, and harmonizing capitalization and punctuation formatting.

### 2.3. Analysis tools and indicators

CiteSpace (version 6.4.R2) was used to construct multiple bibliometric networks, including author, collaboration, institutional collaboration, and national collaboration networks. It was also used for keyword co-occurrence, clustering, and burst analyses; co-citation analysis of cited references and journals; and disciplinary biclustering analysis. The time slice was set to 1 year in this study. The node types were selected as Author, Institution, Country, Keyword, Cited Reference, and Cited Journal, respectively. The g-index, with a scale factor of *k* = 25, was used as the uniform node-selection criterion across all networks, and cluster and timeline views were used for visualization. Clustering quality was assessed using CiteSpace’s modularity *Q* and silhouette *S* scores. A modularity *Q* > 0.3 indicates a valid community structure. A silhouette *S* > 0.5 indicates well-separated, homogeneous clusters. The Bibliometrix R package (developed by Massimo Aria and Corrado Cuccurullo and maintained by K-Synth Srl) was used to analyze annual publication trends, journal contributions, and locally highly cited publications.

### 2.4. Quality control

Two researchers independently screened the titles and abstracts to exclude thematically irrelevant records. Unlike a systematic review, this step aimed only to remove obviously off-topic retrieval noise rather than to assess the methodological quality of individual studies. Any inconsistencies in screening decisions were resolved through discussion with a senior researcher to ensure the reliability of the final dataset. Since this study only analyzed publicly available bibliographic metadata, ethical approval and informed consent were not required.

## 3. Results

### 3.1. Trends in annual publications

Initially, 4409 records were identified, of which 3943 eligible publications were finally included in the analysis, comprising 3283 original articles and 660 reviews. The detailed literature screening process is presented in Figure 1.

**Figure 1.**
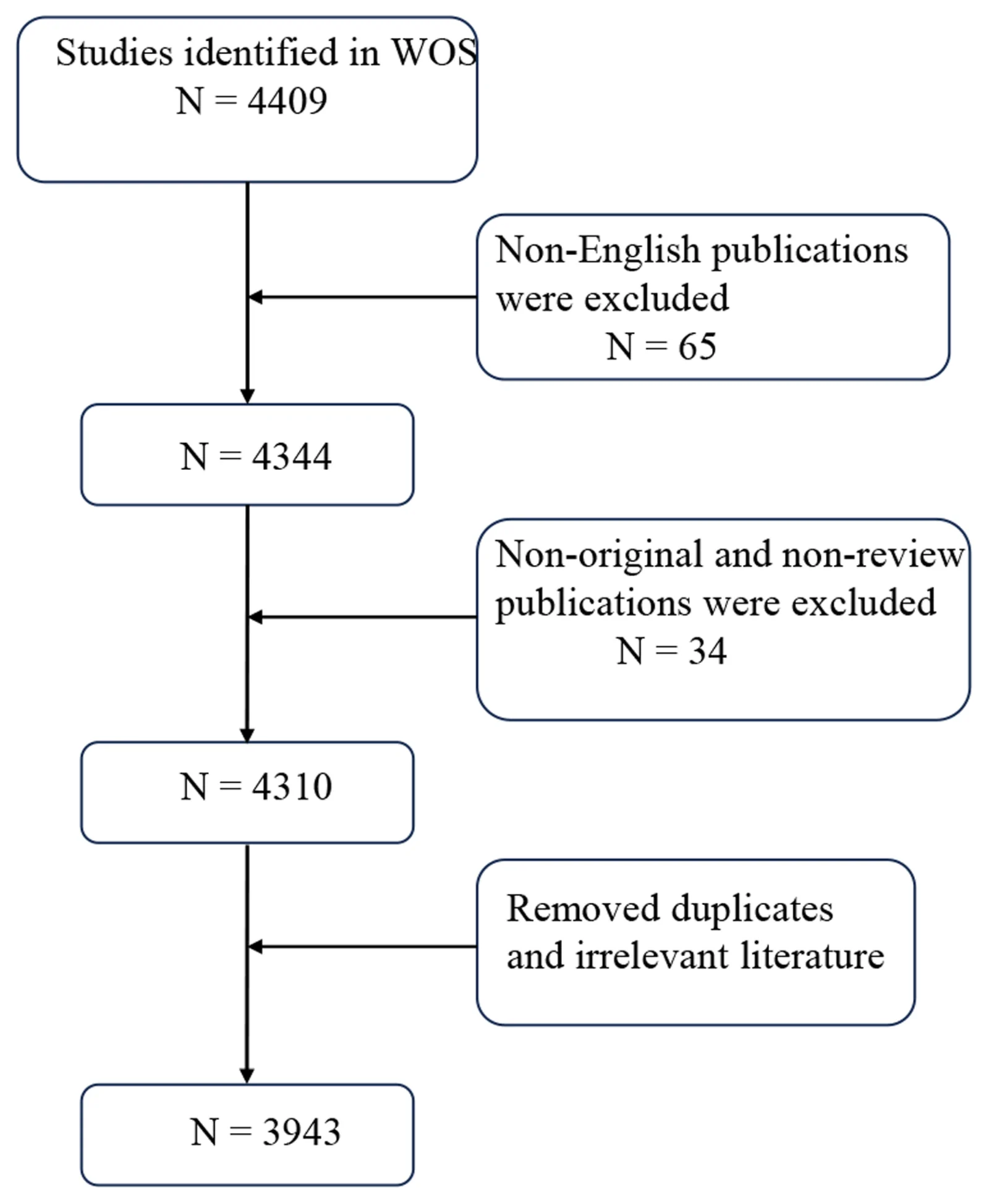
Flow diagram of the literature screening process. WOS = Web of Science.

Figure 2A presents the temporal trends in annual publications. The temporal trend analysis only included publications from 2010 to 2025. Since our literature retrieval was completed on March 29, 2026, incomplete full-year data for 2026 were excluded to avoid bias in comparisons of annual research output. From 2010 to 2025, annual publications concerning olanzapine for schizophrenia initially increased and reached the global peak of 323 in 2013, followed by general downward fluctuations. The publication volume increased from 294 in 2010 to the peak in 2013. A relatively stable low-output plateau occurred from 2017 to 2020, with annual outputs fluctuating mildly between 203 and 206. A secondary peak of 242 was observed in 2021, after which publications declined again to 189 in 2024, followed by a slight rebound to 208 in 2025.

**Figure 2.**
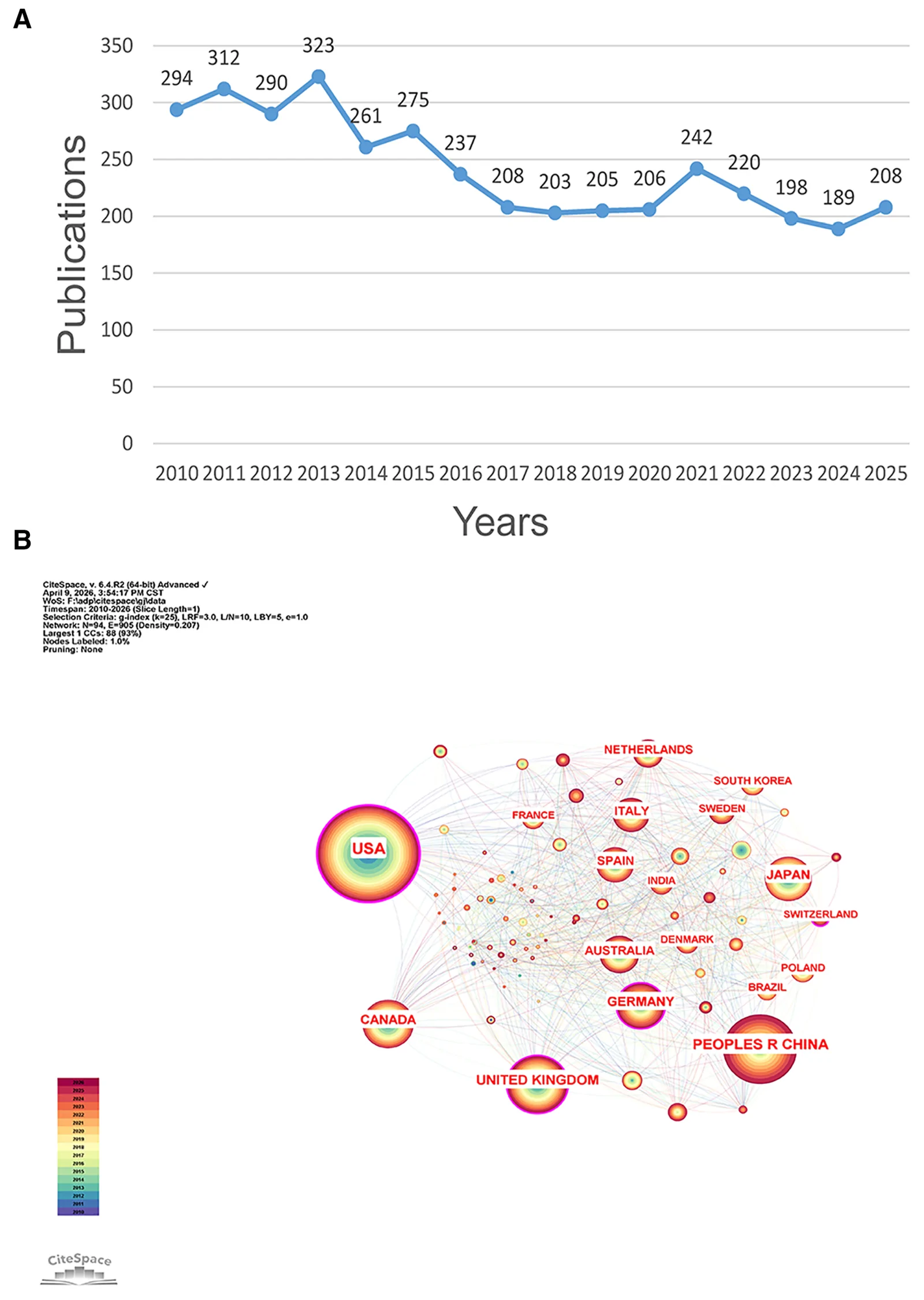
Publication output and global distribution characteristics. (A) Temporal trends in annual publications. (B) Cross-national and regional research distribution.

### 3.2. National/regional collaboration network

Research on olanzapine for treating schizophrenia has been conducted in 94 countries and regions worldwide. Figure 2B illustrates the international collaboration network, exhibiting a distinct core–periphery structure. The size of each node and the density of connections represent regional research output and the level of cross-national cooperation, respectively. Table 1 lists the top 10 countries according to total publication volume.

**Table 1:** Top 10 contributing countries/regions based on publication volume.

Countries	Count	Percentage (%)	Centrality
United States	1212	29.14	0.25
China	666	16.01	0.04
United Kingdom	437	10.51	0.17
Canada	370	8.90	0.09
Germany	331	7.96	0.12
Japan	323	7.77	0.03
Spain	233	5.60	0.05
Australia	223	5.36	0.06
Italy	208	5.00	0.1
Netherlands	156	3.75	0.1

The United States was the most productive country, with 1212 publications (29.14%) and the highest betweenness centrality of 0.25, indicating a prominent position within the international collaboration network. China (666 publications, 16.01%) and the United Kingdom (437 publications, 10.51%) formed the core areas of the network, with large node scales and intensive collaborative ties, and played important roles in driving disciplinary development.

The United Kingdom, Canada, Germany, and Japan each produced more than 300 publications. These countries had high research output and positive betweenness centrality, indicating that they served as important bridges connecting research communities. Spain, Italy, Australia, and New Zealand formed the second collaborative cluster. Stable research output and frequent transnational collaborations have further strengthened the global research collaboration network in this field.

### 3.3. Institutional collaboration network

The institutional collaboration network comprised 581 nodes and 2941 links, with a network density of 0.0175 (Fig. 3A). This suggests that institutional collaboration in this field remains relatively dispersed and has not yet developed a highly integrated global collaboration network.

**Figure 3.**
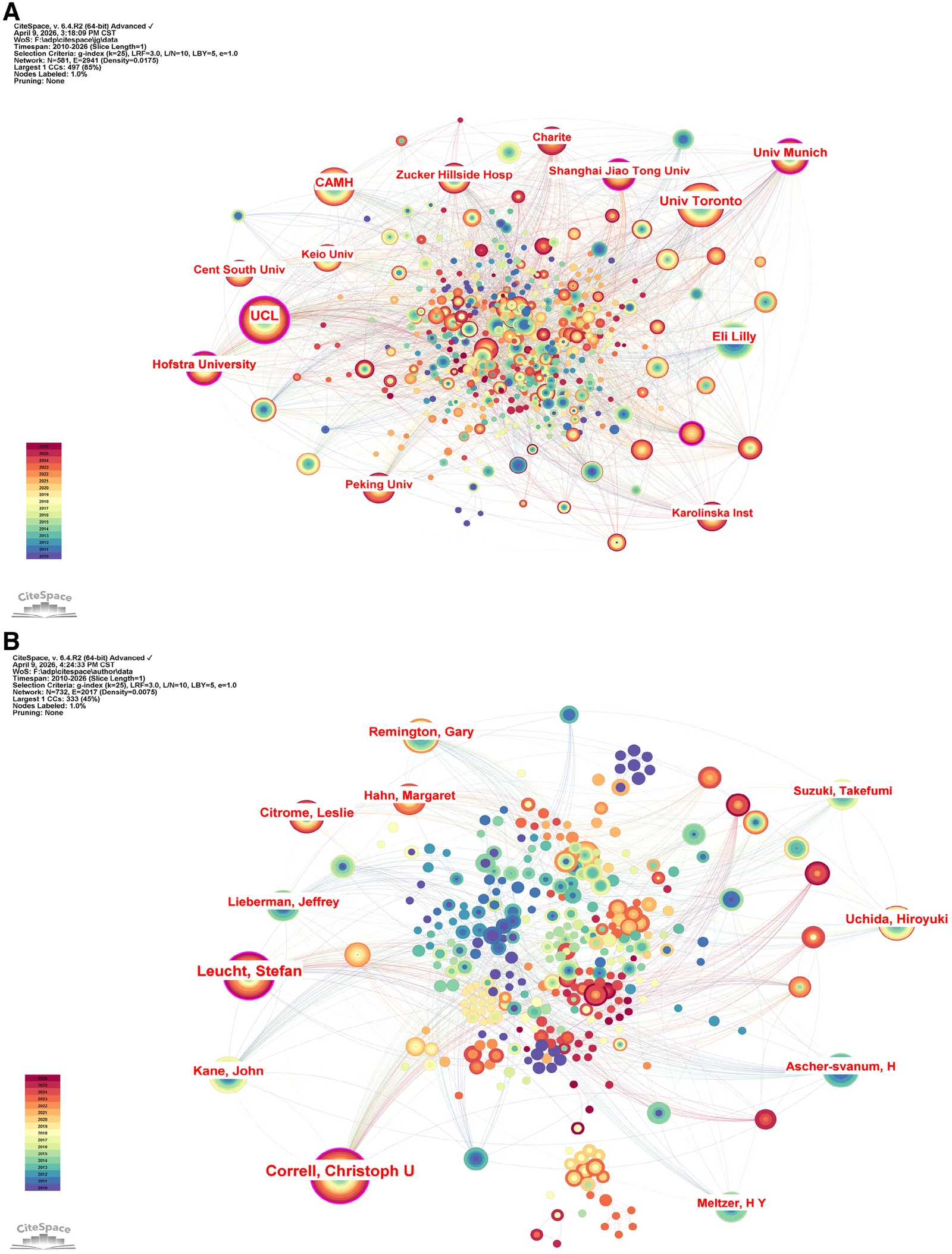
Visualization of institutional and author collaboration networks constructed using CiteSpace. (A) Institutional collaboration network: node size represents the number of publications; line thickness indicates the strength of the collaborative relationship; and node color represents the year of the institution’s first publication. Larger nodes identify highly productive institutions, and hub nodes can be identified by combining this with betweenness centrality. Spatially clustered nodes represent regional collaboration clusters. (B) Author collaboration network: node size represents the number of publications; line thickness indicates the degree of collaboration; and node color represents the year of the author’s first included publication. The clusters of nodes correspond to independent research teams.

Table 2 shows the top 10 institutions by number of publications. Analysis of the 2 bibliometric indicators revealed that University College London (UCL) ranked first globally, with 184 publications, and also possessed the highest betweenness centrality (0.25) in the network, a value 2.3 times higher than that of the University of Munich, which ranked second in betweenness centrality, and was significantly higher than the average value among the top 10 institutions. This combination of high publication output and cross-regional connectivity highlighted its important role as a hub within the collaborative network. The University of Toronto and the Centre for Addiction and Mental Health followed closely behind in terms of publication numbers, but their betweenness centrality was relatively low (0.03–0.09), and their collaborative ties were primarily confined to regional clusters; their role as cross-cluster bridges within this network was weaker than that of UCL.

**Table 2:** Top 10 institutions with respect to the number of documents.

Institutions	Count	Percentage (%)	Centrality
UCL	184	15.97	0.25
Univ Toronto	174	15.10	0.09
CAMH	132	11.46	0.03
Eli Lilly	127	11.02	0.05
Univ Munich	113	9.81	0.11
Hofstra University	92	7.99	0.11
Zucker Hillside Hosp	89	7.73	0.09
Peking Univ	83	7.20	0.07
Shanghai Jiao Tong Univ	80	6.94	0.1
Cent South Univ	78	6.77	0.04

CAMH = Centre for Addiction and Mental Health, UCL = University College London.

Chinese institutions, represented by Peking University and Shanghai Jiao Tong University, ranked among the top 10 globally in publication output, thereby forming a secondary core cluster within the network. However, the betweenness centrality of these institutions generally ranged from 0.04 to 0.10, indicating that there is still room for improvement in their capacity for deep cross-regional and cross-national collaboration. Furthermore, Eli Lilly was the only pharmaceutical company among the top 5 institutions, standing out for its publication volume and network centrality and serving as a key node linking academic and industrial research within the network.

### 3.4. Author collaboration network

The author collaboration network comprised 732 nodes and 2017 links, with a network density of 0.0075 (Fig. 3B). The low network density indicates that collaboration among researchers in this field is generally loose. Small, independent teams primarily conduct research, and large-scale international research consortia have not yet formed.

Table 3 lists the top 10 authors by publication count and their betweenness centrality. Christoph U. Correll and Stefan Leucht ranked first and second, respectively, in publication output, accounting for 37.6% of the combined publications by the top 10 authors. Their betweenness centralities were 0.13 and 0.14, respectively, which were more than 2.5 times the average level of the remaining top 10 authors, indicating that they occupied relatively prominent bridging positions within the collaboration network. Both consistently maintained high levels of research output and cross-team academic engagement, reflecting their active participation and influence within the field’s academic exchange network. The betweenness centrality of the remaining high-output authors mostly ranged from 0.01 to 0.08, indicating that their collaborative influence is largely confined to their research teams.

**Table 3:** Top 10 authors with respect to the number of documents.

Authors	Count	Percentage (%)	Centrality
Christoph U. Correll	103	21.28	0.13
Stefan Leucht	79	16.32	0.14
John Kane	43	8.88	0.08
Gary Remington	41	8.47	0.03
Hiroyuki Uchida	41	8.47	0.02
Leslie Citrome	39	8.06	0.02
H. Ascher-Svanum	38	7.85	0.03
Margaret Hahn	34	7.02	0.03
Jeffrey Lieberman	34	7.02	0.05
H. Y. Meltzer	32	6.61	0.01

### 3.5. Journal distribution and influence analysis

This study systematically analyzed the journal distribution and citation network characteristics of literature on olanzapine treatment for schizophrenia. The analysis was conducted from 3 perspectives: publication trends in core journals, the structure of the co-citation network among journals, and citation-emergence patterns. The relevant results are presented in Figure 4.

**Figure 4.**
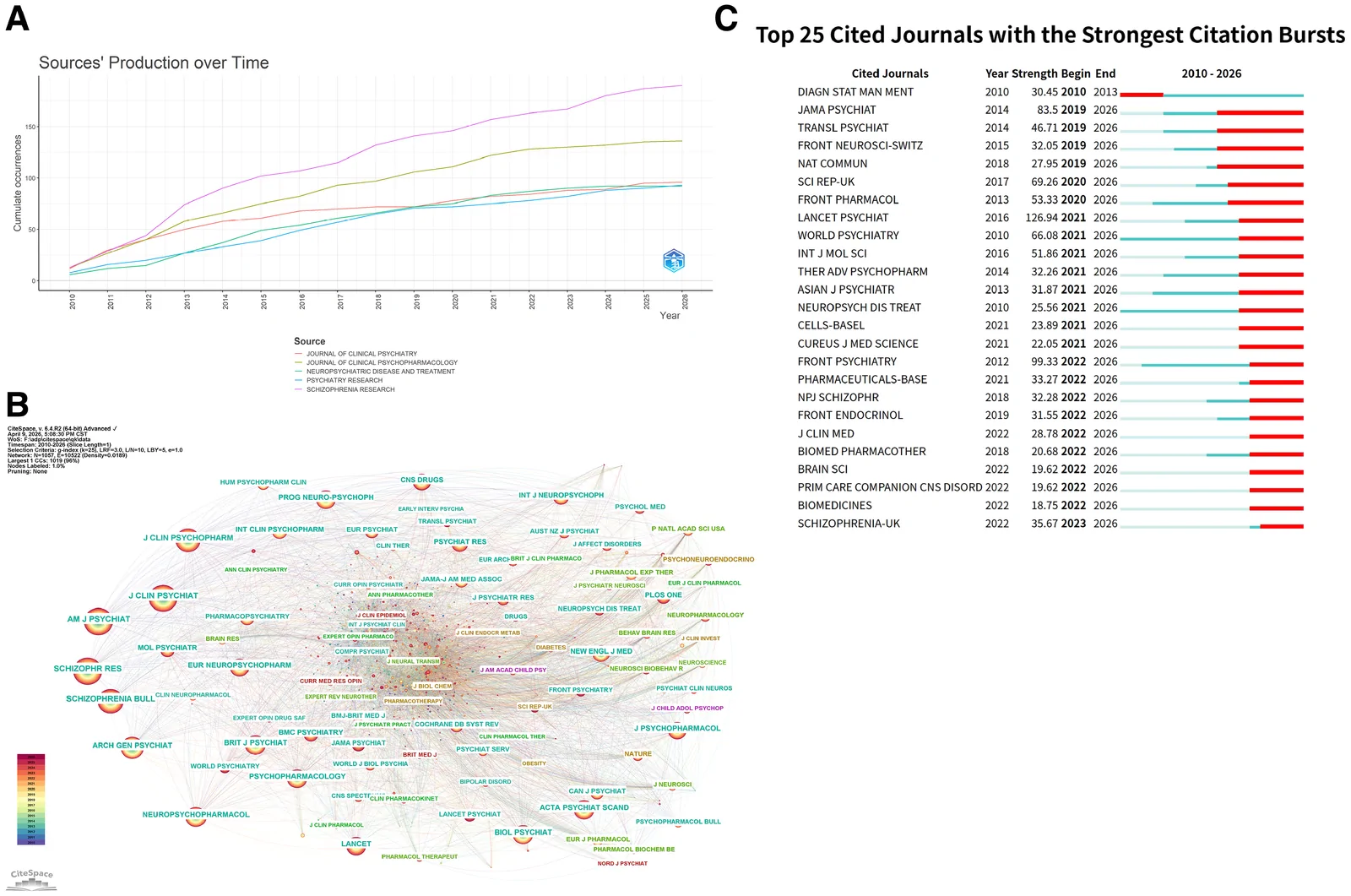
Journal distribution, co-citation network, and citation burst analysis of olanzapine research in schizophrenia. (A) Temporal trends of cumulative publications in core journals, presenting the growth patterns of high-yield journals in the field from 2010 to 2026. (B) Journal co-citation network generated using CiteSpace. The node size represents the co-citation frequency, and the color gradient indicates the publication year of the literature. (C) Top 25 journals with the strongest citation bursts from 2010 to 2026. The red segments represent the burst periods, illustrating the temporal evolution of cutting-edge publication venues in the field.

The included studies were published in 187 academic journals. Figure 4A illustrates the distribution of cumulative publications in core journals. Table 4 lists the top 5 journals by total publication volume, a metric that reflects the scale of research output for each journal. *Schizophrenia Research* ranked first with 190 included articles. With an impact factor (IF) of 3.5 in 2025 and a Journal Citation Reports (JCR) Q1 ranking, the journal has maintained steady growth in publication volume and was the most productive journal in the dataset. This was followed by *Journal of Clinical Psychopharmacology* (135 articles, IF = 2.8, Q2), *The Journal of Clinical Psychiatry* (96 articles, IF = 4.6, Q1), *Psychiatry Research* (93 articles, IF = 3.9, Q1), and *Neuropsychiatric Disease and Treatment* (93 articles, IF = 2.9, Q2).

**Table 4:** The 5 leading journals in publication volume.

Journal	Publication	IF (2025)	JCR (2025)
*Schizophrenia Research*	190	3.5	Q1
*Journal of Clinical Psychopharmacology*	135	2.8	Q2
*The Journal of Clinical Psychiatry*	96	4.6	Q1
*Psychiatry Research*	93	3.9	Q1
*Neuropsychiatric Disease and Treatment*	93	2.9	Q2

IF = impact factor, JCR = Journal Citation Reports.

Table 5 summarizes the top 5 journals by total citation count, reflecting the level of citation activity within the field. Several JCR Q1 journals (*The American Journal of Psychiatry*, *Schizophrenia Research*, and *The Journal of Clinical Psychiatry*) exhibited high citation activity, with each having a total citation count exceeding 2000. The journal citation network (Fig. 4B) showed that the nodes representing these journals were larger and the connections between them were tighter, indicating strong co-citation relationships among them.

**Table 5:** Top 5 journals by co-citation frequency.

Journal	Co-citations	IF (2025)	JCR (2025)
*The American Journal of Psychiatry*	2802	14.7	Q1
*Schizophrenia Research*	2790	3.5	Q1
*The Journal of Clinical Psychiatry*	2606	4.6	Q1
*Schizophrenia Bulletin*	2250	4.8	Q1
*Journal of Clinical Psychopharmacology*	2067	2.8	Q2

IF = impact factor, JCR = Journal Citation Reports.

Figure 4C presents the citation emergence results calculated using the Kleinberg emergence algorithm, reflecting the temporal dynamics of citation attention received by different journals. Established journals, such as *JAMA Psychiatry* and *World Psychiatry*, consistently attracted scholarly attention throughout the study period. In contrast, some journals have displayed a rapid rise in citation attention in recent years. *The Lancet Psychiatry* achieved an emergence intensity of 126.94 from 2021 to 2026, while *Frontiers in Psychiatry* reached 99.33 during the same period; both journals exhibited steadily increasing citation attention in this field.

### 3.6. Reference co-citation analysis

Local citation frequency analysis revealed that the core knowledge base of the field is composed of a series of highly cited publications (Fig. 5A). Among them, the study by Lieberman JA et al (2005) in *The New England Journal of Medicine*, entitled “Effectiveness of antipsychotic drugs in patients with chronic schizophrenia,” had the highest local citation frequency (653 times), providing key long-term efficacy evidence for the clinical use of olanzapine. Leucht et al published 2 landmark meta-analyses in *The Lancet* (2009 and 2013), entitled “Second-generation versus first-generation antipsychotic drugs for schizophrenia: a meta-analysis” and “Comparative efficacy and tolerability of 15 antipsychotic drugs in schizophrenia: a multiple-treatments meta-analysis.” These works have been cited 265 and 372 times, respectively, and systematically quantified the efficacy and safety profiles of olanzapine relative to other antipsychotics, laying a foundational framework for comparative effectiveness research in this area. Together, these highly cited studies constitute the core knowledge base underpinning olanzapine research for schizophrenia.

**Figure 5.**
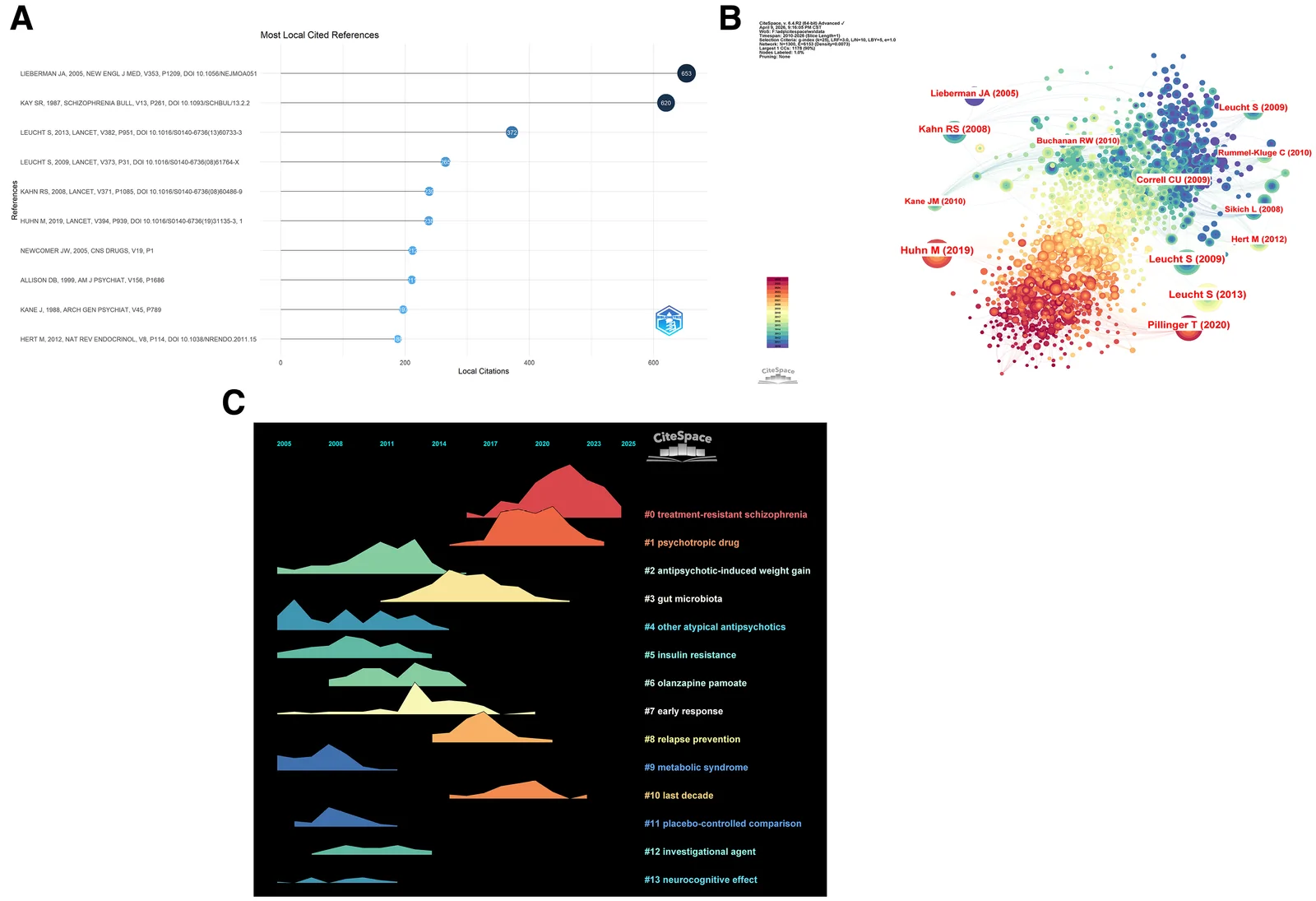
Bibliometric mapping of reference features and thematic evolution in the field of olanzapine for treating schizophrenia. (A) Ranking of the top 10 locally cited references based on internal citation frequency in the dataset. (B) Reference co-citation network constructed using CiteSpace; node diameter reflects citation popularity, and color variation denotes the publication timeline of the cited literature. (C) Thematic cluster labels generated from reference co-citation clustering analysis using CiteSpace.

Figure 5B shows the reference co-citation network constructed by CiteSpace. Document co-citation network analysis revealed that the study by Leucht S et al (2009), published in *The Lancet*, entitled “Second-generation versus first-generation antipsychotic drugs for schizophrenia: a meta-analysis,” systematically compared the efficacy and safety of second-generation and first-generation antipsychotics and confirmed that olanzapine presented significantly better efficacy than first-generation agents, establishing the gold standard for comparative effectiveness research in this field. In 2013, the same group published a landmark network meta-analysis in *The Lancet* entitled “Comparative efficacy and tolerability of 15 antipsychotic drugs in schizophrenia: a multiple-treatments meta-analysis,” which included 15 antipsychotic drugs and verified that olanzapine ranked among the top in efficacy, with systematic quantification of efficacy and tolerability across medications. Furthermore, the study by Correll CU et al (2009) in *JAMA*, entitled “Cardiometabolic risk of second-generation antipsychotic medications during first-time use in children and adolescents,” was the first large-scale investigation to assess metabolic risks associated with initial olanzapine use in children and adolescents, raising extensive attention to the safety profile of olanzapine. Collectively, these high-impact landmark studies constitute the core knowledge base for research on olanzapine in the treatment of schizophrenia.

The CiteSpace document co-citation clustering labels (Fig. 5C) indicated that the knowledge base in the research field of olanzapine for schizophrenia could be divided into 3 thematic categories. One research category addressed therapeutic effects and fundamental pharmacological mechanisms, which covered Cluster #4 concerning other atypical antipsychotics, Cluster #11 related to placebo-controlled comparative trials, as well as Cluster #13 focusing on neurocognitive impacts. Another category was dedicated to metabolic side effects and relevant clinical intervention strategies. It consisted of multiple thematic clusters, including weight gain triggered by antipsychotic medications in Cluster #2, insulin resistance in Cluster #5, metabolic syndrome issues in Cluster #9, olanzapine pamoate application in Cluster #6, early therapeutic response assessment in Cluster #7, relapse prevention work in Cluster #8, and studies on investigational agents in Cluster #12. The last category corresponded to newly developing research hotspots and in-depth mechanism exploration. This section contained Cluster #0 for treatment-resistant schizophrenia, Cluster #1 about psychotropic drugs, Cluster #3 associated with gut microbiota, and Cluster #10 summarizing research achievements made over the past 10 years.

### 3.7. Keyword co-occurrence and research hotspot analysis

The keyword co-occurrence network (Fig. 6A) intuitively reveals the core knowledge structure and thematic correlations of studies on olanzapine for schizophrenia. Olanzapine and schizophrenia were the largest nodes with the highest centrality values, constituting the core of research in this field.

**Figure 6.**
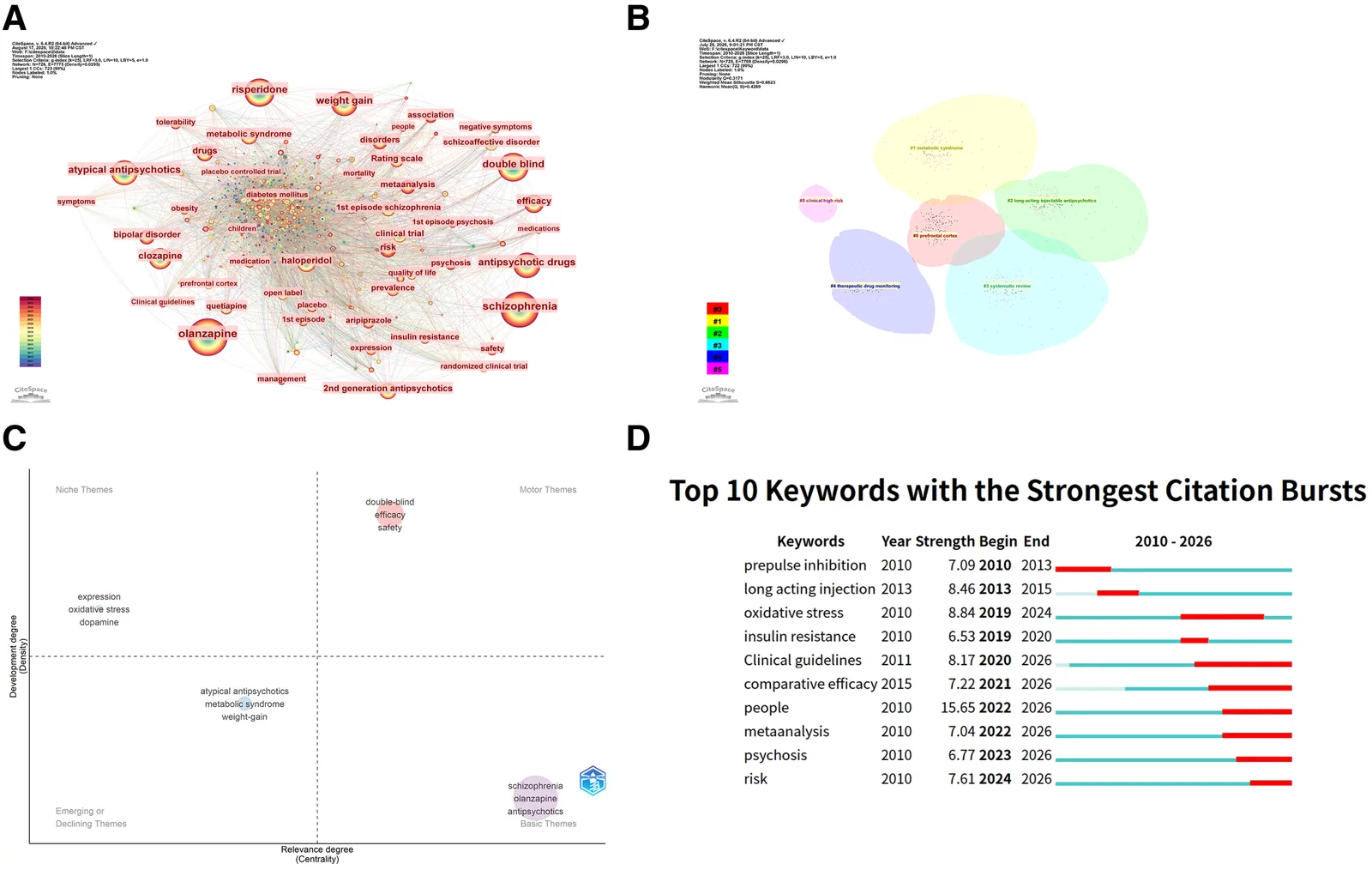
Keyword bibliometric analysis of studies on olanzapine for treating schizophrenia. (A) Keyword co-occurrence network diagram. The node size represents the frequency of keyword occurrence, and the connecting lines indicate co-occurrence relationships between paired keywords. (B) Keyword clustering map generated using CiteSpace. (C) Strategic diagram of research themes. The X-axis denotes centrality (theme relevance to the whole field), and the Y-axis denotes density (internal maturity of each theme). (D) Top 10 keywords with the strongest citation bursts from 2010 to 2026.

Cluster analysis was further performed on the keyword network (Fig. 6B), with a modularity *Q* of 0.3171 and weighted mean silhouette *S* of 0.6623. The 2 metrics indicated clear separation among the clusters and high internal homogeneity within each cluster. The network formed 6 keyword clusters, with the following cluster labels: Cluster #0, prefrontal cortex; Cluster #1, metabolic syndrome; Cluster #2, long-acting injectable antipsychotics; Cluster #3, systematic review; Cluster #4, therapeutic drug monitoring; and Cluster #5, clinical high risk.

The strategic coordinate analysis (Fig. 6C) categorized all research themes into 4 quadrants according to thematic centrality (horizontal axis) and research density (vertical axis). Centrality represents the interconnection of a theme within the entire research network, whereas density reflects the maturity of the thematic research system. Themes in the lower-right quadrant were classified as basic themes, mainly olanzapine, schizophrenia, and antipsychotic drugs, forming the fundamental disciplinary framework. Themes in the upper-right quadrant are motor themes with robust development momentum, including efficacy evaluation, safety assessment, and double-blind trial design. These themes feature strong cross-theme connections and mature research systems and contribute to the development of the field. The upper-left quadrant consists of niche themes such as oxidative stress, dopamine regulation, and gene expression. Themes in the lower-left quadrant (atypical antipsychotics, metabolic syndrome, and weight gain) exhibit low centrality and immature research systems, suggesting declining research attention and a need for further systematic investigation.

Keyword burst detection (Fig. 6D) identifies evolving research frontiers and temporal dynamics. Strong citation bursts were observed for multiple keywords between 2021 and 2026, including people, clinical guidelines, comparative efficacy, meta-analysis, psychosis, and risk. This temporal transition suggested that research priorities had shifted from early basic mechanistic exploration to population-based real-world clinical research, the formulation of evidence-based clinical guidelines, comparative evaluation of multiple treatment regimens, and long-term adverse risk management. The keyword “people” had the highest burst strength (15.65), highlighting that individualized medication strategies and population-specific pharmacotherapy characteristics have become a central research hotspot. Ongoing updates to clinical guidelines and comparative efficacy demonstrate an urgent need for high-quality clinical evidence to standardize and optimize olanzapine treatment pathways. Continuous bursts of risk and psychosis further suggest growing attention to the use of olanzapine across schizophrenia spectrum disorders and long-term safety risk assessment.

### 3.8. Journal dual-map overlay analysis: disciplinary distribution and knowledge flow characteristics

The journal dual-map overlay (Fig. 7) intuitively reveals the disciplinary knowledge flow and interdisciplinary characteristics in the field of olanzapine for schizophrenia, showing a pattern characterized by dual core sources and multiple knowledge-output pathways. The cited journal clusters on the left indicate that domain knowledge originated primarily from 2 core disciplines. The first is clinical medicine, which provides fundamental evidence-based support for the clinical efficacy and safety of olanzapine. The second is molecular biology and immunology, which provide a theoretical basis for exploring drug mechanisms and therapeutic targets. The citing journal clusters on the right demonstrate that research findings were primarily published in 2 major disciplinary domains: health/nursing/medicine and molecular biology/genetics, reflecting, respectively, the optimization of clinical applications and the exploration of translational mechanisms. The connecting lines in the middle further reveal 3 major knowledge-flow pathways: the direct knowledge flow from clinical medicine to health/nursing/medicine, the extension of knowledge from molecular biology to molecular genetics and neuroscience, and the expansion from clinical medicine and psychology to psychosocial rehabilitation. This framework reveals a knowledge-transmission chain of evidence-based clinical practice, basic translational research, and psychosocial rehabilitation. These findings provide a visual framework for understanding the interdisciplinary integration and developmental logic of olanzapine research for treating schizophrenia.

**Figure 7.**
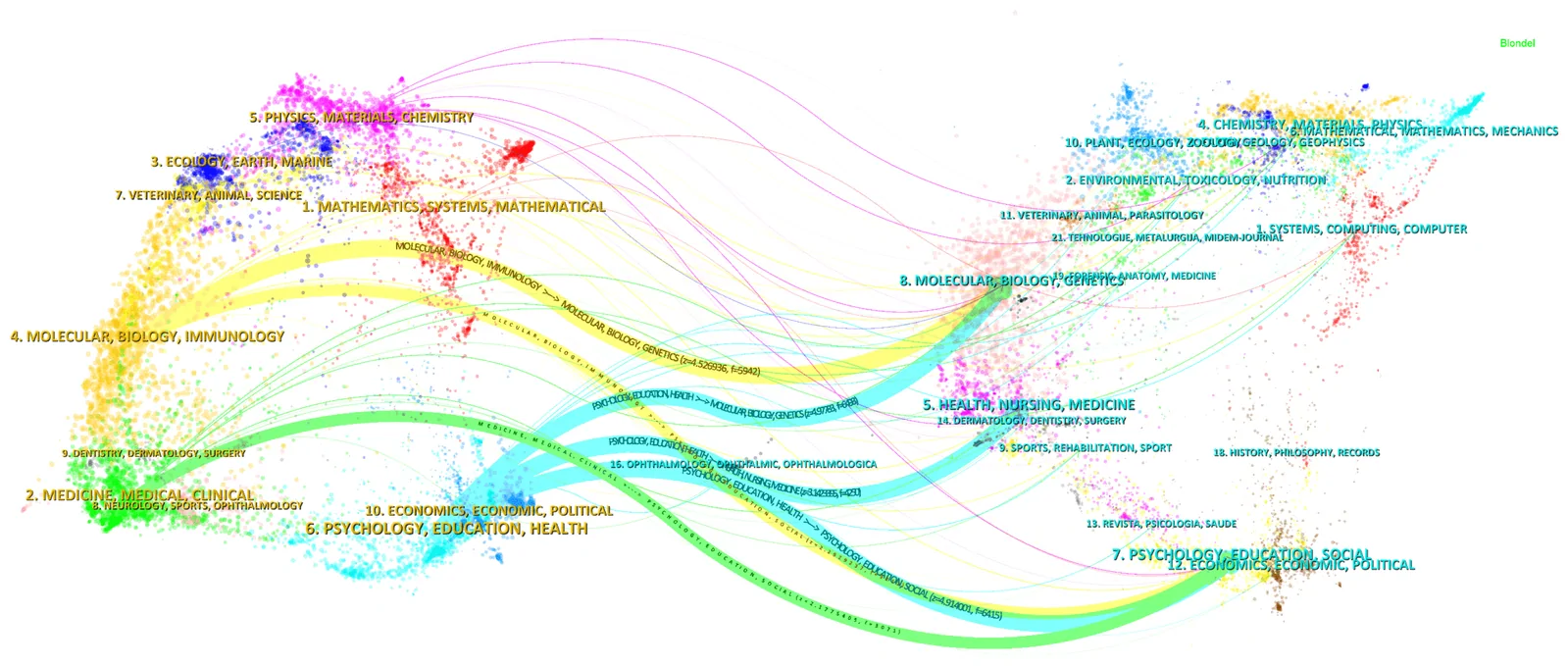
Journal dual-map overlay displaying the pattern of disciplinary knowledge flow. The left side represents the cited journals as the knowledge source, while the right side represents the citing journals for knowledge output. The thickness of the connecting lines indicates the intensity of interdisciplinary associations.

## 4. Discussion

### 4.1. Analysis of annual publication trends

Fluctuations in the volume of global publications on olanzapine treatment for schizophrenia over the past 15 years objectively reflect the phased shifts in core research directions within this field, which is a typical developmental pattern of a mature discipline in psychopharmacology.^[22]^ Based on bibliometric characteristics, the entire research trajectory can be divided into 4 distinct developmental phases, each with markedly different academic focuses.

The period from 2010 to 2013 marked a phase of rapid research expansion. During this stage, a large number of high-evidence-level network meta-analyses and multicenter clinical trials were published in succession, with the academic focus concentrated on validating the acute-phase efficacy of olanzapine. A multidrug comparative meta-analysis published by Leucht’s team in 2013 established the efficacy profile of olanzapine relative to other antipsychotics, spurring a large number of short-term controlled clinical studies and causing the annual publication volume to peak in 2013.^[23]^

After reaching its peak in 2013, publication output declined in 2014 and then entered a stable plateau phase from 2015 to 2020. Although the annual number of publications remained steady at over 200, an analysis of the temporal patterns of citation clusters revealed a structural shift in the discipline’s research focus.

The years 2021 to 2023 witnessed a second wave of publication growth, forming a secondary research peak driven by 2 major industry events: the approval and launch of the olanzapine/samidorphan combination formulation and the revision of global psychiatric diagnostic and treatment guidelines. The new combination formulation spurred a concentrated output of research balancing efficacy and metabolic risks, while the need for guideline updates also led to a significant increase in literature comparing real-world efficacy.^[24]^ After 2024, the annual number of publications fluctuated slightly but remained at a moderate level overall, marking the discipline’s entry into a phase of refined and mature development.

### 4.2. Characteristics of the national/regional collaboration landscape

Globally, research on olanzapine exhibited a typical core–periphery geographical distribution. The United States has long occupied a central position in the collaboration network. Supported by national mental health research funding, pharmaceutical industry collaboration, and landmark large-scale clinical studies such as Clinical Antipsychotic Trials of Intervention Effectiveness, the United States has contributed substantially to the field’s core knowledge and citation structure and has long dominated the design of high-level clinical trials and the development of evidence-based knowledge.^[25]^ With its strong geographical and institutional connections, the United Kingdom has become a key hub for academic exchange between Europe and the United States. Canada, Germany, and Japan each published more than 300 papers and engaged in frequent transnational collaboration, collectively forming a secondary core collaborative cluster; Spain, Italy, Australia, and New Zealand constituted a secondary collaborative tier, with their stable research output and routine transnational collaboration further enhancing the global research collaboration system. China has demonstrated remarkable growth in scientific research in recent years, but its role as an intermediary hub remains limited, and there are significant shortcomings in transnational academic collaboration.

### 4.3. Characteristics of institutional collaboration

The density of the institutional collaboration network is relatively low, indicating that collaborative relationships among institutions remain fragmented and have not yet developed into a unified, large-scale global research network, highlighting a common limitation of academic collaboration in psychopharmacology.

UCL ranked first in both publication output and betweenness centrality, and its exceptionally strong intercontinental connectivity positioned it as a core hub for international academic exchange. Institutions such as the University of Toronto and Centre for Addiction and Mental Health have substantial publication volumes, but their betweenness centrality ranges only from 0.03 to 0.09. Their collaborative relationships are confined to their respective regions and exhibit relatively limited cross-network connectivity.

Eli Lilly is a leading pharmaceutical company in terms of its publication volume. As the originator of olanzapine, it has established a complete R&D chain spanning basic pharmacology, multi-formulation development, and global real-world clinical validation.^[17]^ Meanwhile, the olanzapine/samidorphan combination developed by Alkermes further advances the optimization of metabolic risk; following its market launch, the combination has also been the subject of numerous comparative studies evaluating efficacy and metabolic safety.^[16]^ Top domestic universities, such as Peking University and Shanghai Jiao Tong University, have ranked among the top 10 globally, forming stable local research clusters; however, their betweenness centrality values ranged from 0.04 to 0.10, indicating insufficient capacity for in-depth transnational collaboration.

### 4.4. Characteristics of the author collaboration network

The author collaboration network exhibited relatively low overall density, suggesting that research in this field was primarily conducted by small, independent research groups, with few large-scale, transnational collaborative research teams. Christoph U. Correll and Stefan Leucht ranked among the top in both publication output and betweenness centrality; the highly cited meta-analyses and metabolic safety cohort studies they published served as key nodes linking disparate research teams worldwide.^[23,26]^ The remaining high-output authors generally exhibited low betweenness centrality, with academic exchanges largely confined to their own teams and limited cross-team academic collaboration.

### 4.5. Characteristics of output and impact of core journals

Analysis of journal-level citation characteristics reveals the core position of several authoritative journals within this knowledge domain. High total citation counts endow *The American Journal of Psychiatry*, *Schizophrenia Research*, and *The Journal of Clinical Psychiatry* with a foundational role in constructing the disciplinary knowledge base. The large node size and dense interconnections observed in the co-citation network reflect close intellectual exchange and frequent knowledge intersection across these core publications. Iconic top-tier journals, including *JAMA Psychiatry* and *World Psychiatry*, maintained persistent disciplinary influence over the investigated period. After 2021, *The Lancet Psychiatry* and *Frontiers in Psychiatry* gained growing scholarly recognition, which coincided with the field’s evolving research priorities toward real-world evidence and precision-oriented investigations.

For cross-journal comparison, this study adopted a unified edition of JCR quartiles and IFs. This is a conventional approach in bibliometric journal-metric analysis and minimizes confounding bias caused by year-to-year fluctuations in journal metrics.^[27]^

### 4.6. Evolution of research themes based on co-citation, keyword clustering, and emergence analysis

By integrating multiple bibliometric maps, including co-citation networks, keyword clustering, strategic coordinates, and keyword emergence, we traced the evolution of olanzapine research. The research focus has gradually moved beyond isolated acute-phase efficacy validation toward diversified research priorities covering clinical safety management, individualized treatment strategies, and real-world-oriented evidence generation. As reflected by citation-cluster temporal features, investigators have placed increasing emphasis on long-term follow-up and interventions for metabolic adverse reactions.^[28]^ Keyword-emergence patterns further reveal that population stratification and individualized dosing have become mainstream research priorities in recent years, whereas publications addressing basic pharmacodynamic mechanisms account for a shrinking proportion of the literature.^[29]^

First, clusters related to weight gain, insulin resistance, and metabolic syndrome in the co-citation network indicate that the management of metabolic risk emerged as a distinct research theme after 2015. This cluster exhibits strong internal connectivity but weak links to early clusters focused on efficacy evaluation, suggesting that efficacy optimization and interventions for metabolic adverse effects have gradually diverged into 2 parallel research tracks. This cluster maintained stable bibliometric activity across the observation period, indicating sustained scholarly attention toward antipsychotic-related metabolic adverse-effect management.^[30]^

Second, the keyword “people” exhibited the highest emergence intensity across the entire domain. Combined with the results of the independent “therapeutic drug monitoring” cluster, these findings suggest that personalized dosing has been a cutting-edge focus since 2021. The persistent prominence of keywords such as “clinical guidelines” and “efficacy comparisons” reflects the urgent need for high-quality, evidence-based data to standardize clinical dosing regimens. The strategic coordinate map identified personalized dosing as a dynamic theme with high centrality and maturity, reflecting its prominent status within the existing research landscape.

Third, the sustained prominence of keywords, such as “systematic reviews” and “long-acting injectables,” aligns with the research direction toward real-world effectiveness comparisons. By leveraging routine clinical cohort data to supplement evidence on relapse prevention and the long-term safety of long-acting olanzapine, these studies support updates to clinical practice guidelines.^[31]^ In the lower-left corner of the strategic coordinate map, themes related to metabolic syndrome and weight gain exhibited low centrality and weak cluster maturity. These findings suggest that research interest in this area may be declining.

## 5. Limitations of the study

First, the literature was retrieved only from the Web of Science Core Collection and not from PubMed, Scopus, or Embase. This limitation has been discussed in previous database comparison studies.^[32]^ PubMed does not provide the same integrated citation data required for co-citation and burst analyses used in this study. Scopus may have limitations in retrospective citation coverage before 2000, and Embase primarily collects European pharmaceutical trials and does not provide standardized full citation datasets. The Web of Science Core Collection provides consistent, long-term citation archives and is widely used as a major source for bibliometric research; however, the single-database strategy may introduce selection bias by missing regional journals and region-specific clinical trials. Nevertheless, high-quality English-language core literature indexed in the Web of Science Core Collection can largely represent mainstream international research trends concerning olanzapine, and this limitation is unlikely to alter our major conclusions.

Second, only English-language original articles and reviews were included, leading to linguistic and geographical biases. Third, the retrieval cutoff date was March 29, 2026; papers published after this date were excluded, and citation lag may underestimate the impact of newly published works. Fourth, self-citations were retained during raw data processing, which may have inflated citation frequencies and centrality measures of certain authors and institutions. Fifth, all visualizations and quantitative results relied on the default algorithmic settings of CiteSpace and Bibliometrix, which may have introduced software-specific bias. Additionally, conference abstracts, editorial letters, and gray literature were excluded during screening, which may further limit the comprehensiveness and generalizability of the findings.

## 6. Conclusion

This bibliometric study mapped the knowledge structure and temporal evolution of olanzapine-for-schizophrenia research. Unlike conventional clinical reviews that mainly synthesize efficacy and safety evidence, the present work provides a holistic bibliometric overview of publication trends, international-institutional collaboration patterns, and thematic evolution in this field.

The United States, China, and the United Kingdom dominate global research output, and UCL acts as a prominent collaborative hub. Christoph U. Correll and Stefan Leucht represent key scholarly contributors, and *Schizophrenia Research* is one of the major active journals.

Our analysis reveals distinct phased shifts in research priorities: early studies centered on acute-phase efficacy and antipsychotic head-to-head comparisons; following confirmation of olanzapine’s efficacy, research expanded toward metabolic-risk management. Recent research frontiers point to population-tailored personalized dosing and real-world evidence supporting clinical guideline updates.

Beyond confirming previously reported clinical research hotspots, this study quantitatively identifies the cross-national collaboration landscape, the bridging-node role of specific institutions and authors, and the temporal divergence between efficacy-focused and metabolic-safety-oriented research clusters. These findings complement existing clinical reviews by offering a quantitative, network-based perspective on how this research domain has evolved.

## Author contributions

**Conceptualization:** Na Shu, Lin Wang, Hai Ming Tang.

**Data curation:** Na Shu, Lin Wang, Yan Gou, Min Zhao, Hai Ming Tang.

**Formal analysis:** Na Shu, Lin Wang, Ling Xin Wang, Yan Gou, Hai Ming Tang.

**Investigation:** Lin Wang, Min Zhao.

**Methodology:** Lin Wang, Ling Xin Wang, Yan Gou, Hai Ming Tang.

**Project administration:** Na Shu, Lin Wang, Yan Gou, Min Zhao, Hai Ming Tang.

**Resources:** Na Shu, Ling Xin Wang, Yan Gou, Min Zhao.

**Software:** Na Shu, Lin Wang, Ling Xin Wang, Yan Gou, Min Zhao, Hai Ming Tang.

**Supervision:** Na Shu, Lin Wang, Ling Xin Wang, Yan Gou, Min Zhao, Hai Ming Tang.

**Validation:** Na Shu, Lin Wang, Ling Xin Wang, Yan Gou, Min Zhao.

**Visualization:** Na Shu, Ling Xin Wang, Hai Ming Tang.

**Writing – original draft:** Na Shu.

**Writing – review & editing:** Hai Ming Tang.


