# Supplementary material for: Research trends in olanzapine in treating schizophrenia from 2010 to 2026: A bibliometric analysis based on the Web of Science Core Collection
Source: Medicine (Baltimore). 2026 Sep 25;105(39):e50747. doi: 10.1097/MD.0000000000050747 (PMC13619229; doi:10.1097/MD.0000000000050747)
Supplement: Supplementary file 1 [file medi-105-e50747-s001.docx]

Database: Web of Science Core Collection, All Editions

Retrieval date: March 29, 2026

Time span: [2010–2026]

Language restriction: English only.

Included document types: Original articles, reviews.

Supplementary Table S1. Full search syntax used in Web of Science Core Collection.

| Search Step | Complete WOS retrieval query |
| --- | --- |
| #1  #2  #3 | TS=olanzapine OR TI=olanzapine OR AB=olanzapine OR AK=olanzapine  TS=schizophrenia OR TI=schizophrenia OR AB=schizophrenia OR AK=schizophrenia  #1 AND #2 |

TS: Topic, TI: Title, AB: Abstract, AK: Author Keywords.
